# Feature integration and object representations along the dorsal stream visual hierarchy

**DOI:** 10.3389/fncom.2014.00084

**Published:** 2014-08-05

**Authors:** Carolyn Jeane Perry, Mazyar Fallah

**Affiliations:** ^1^Visual Perception and Attention Laboratory, School of Kinesiology and Health Science, York UniversityToronto, ON, Canada; ^2^Centre for Vision Research, York UniversityToronto, ON, Canada; ^3^Departments of Biology and Psychology, York UniversityToronto, ON, Canada; ^4^Canadian Action and Perception Network, York UniversityToronto, ON, Canada

**Keywords:** feature integration, dorsal pathway, object representation, motion processing, decision making

## Abstract

The visual system is split into two processing streams: a ventral stream that receives color and form information and a dorsal stream that receives motion information. Each stream processes that information hierarchically, with each stage building upon the previous. In the ventral stream this leads to the formation of object representations that ultimately allow for object recognition regardless of changes in the surrounding environment. In the dorsal stream, this hierarchical processing has classically been thought to lead to the computation of complex motion in three dimensions. However, there is evidence to suggest that there is integration of both dorsal and ventral stream information into motion computation processes, giving rise to intermediate object representations, which facilitate object selection and decision making mechanisms in the dorsal stream. First we review the hierarchical processing of motion along the dorsal stream and the building up of object representations along the ventral stream. Then we discuss recent work on the integration of ventral and dorsal stream features that lead to intermediate object representations in the dorsal stream. Finally we propose a framework describing how and at what stage different features are integrated into dorsal visual stream object representations. Determining the integration of features along the dorsal stream is necessary to understand not only how the dorsal stream builds up an object representation but also which computations are performed on object representations instead of local features.

## Introduction

Classically, visual processing from the retina onwards is described as following two general principles. One, the processing of different types of visual information is anatomically segregated into two visual streams, and two, each stream is comprised of hierarchical processing where each stage builds upon the previous stage, becoming increasingly more complex. In the ventral pathway this ultimately results in an ability to recognize objects in spite of changes in the surrounding environment or changes in certain object features (i.e., position, orientation, viewing angle, size, etc). In the dorsal pathway this hierarchical processing produces computations of complex motion of objects within the environment around us, either as we are stationary or moving through that environment. Because of this functional separation, there are many models of object representation in the ventral stream (see Peissig and Tarr, 2007 for a review) and many models of motion processing in the dorsal stream (for reviews see Burr and Thompson, 2011; Nishida, 2011), but motion processing research has been mostly devoid of investigations as to the nature or existence of object representations in the dorsal stream. In fact, the vision for action theory of dorsal stream function (Goodale and Milner, 1992; Goodale, 2008, 2013) would suggest that even though there might not be an internal representation of the object as a whole (see Farivar, 2009 for an alternative view), there are representations of features of an object that are relevant for action in real time. Evidence for this comes from spared functions in visual agnosia wherein damage to the ventral pathway eliminates the ability to recognize objects but spares scaling and orientation of the hand when grasping objects (Goodale et al., 1991, 1994; Milner et al., 2012). In addition, parietal regions of the dorsal pathway involved in reaching and grasping show selectivities for the orientation, shape and size of objects (Taira et al., 1990; Gallese et al., 1994; Murata et al., 2000; Fattori et al., 2005).

More recently, investigations into cross-talk between the two visual streams suggest that there are object representations in the dorsal stream (Schiller, 1993; Sereno and Maunsell, 1998; Tsutsui et al., 2001; Sereno et al., 2002; Peuskens et al., 2004; Durand et al., 2007; Lehky and Sereno, 2007; Wannig et al., 2007; Konen and Kastner, 2008; Tchernikov and Fallah, 2010; Perry and Fallah, 2012). It is important to note however, that this object representation would not necessarily be one that gives rise to object recognition, as in the ventral stream. For example, it has been shown that recognition of objects constructed from coherently moving dots (structure-from-motion) is severely impaired in visual agnosiacs (Huberle et al., 2012). These cross-talk studies suggest however, that the motion computations that occur within the dorsal stream can benefit from an intermediate object representation that includes different features of the object. This intermediate object representation would allow for selection of one moving object over others contained within the visual field as seen with flankers and crowding (Livne and Sagi, 2007; Malania et al., 2007; Sayim et al., 2008; Manassi et al., 2012; Chicherov et al., 2014), and superimposed surfaces (Valdes-Sosa et al., 1998; Rodríguez et al., 2002; Mitchell et al., 2003; Reynolds et al., 2003; Stoner et al., 2005; Fallah et al., 2007; Wannig et al., 2007).

In this review we will first give a brief overview of the hierarchical nature of feature processing in both the ventral and dorsal pathways. Various models of the ventral stream have been proposed wherein each integrates features to build up an object representation (scale invariant feature transform (SIFT): Lowe, 1987; Neocognitron: Fukushima, 1975; hierarchical model and X (HMAX): Riesenhuber and Poggio, 1999, and others. For review see Poggio and Ullman, 2013), often based on behavioral and neurophysiological studies (Cowey and Weiskrantz, 1967; Gross et al., 1971, 1972; Dean, 1976; Marr and Nishihara, 1978; Biederman, 1987; Biederman and Cooper, 1991). However, the dorsal stream has generally been relegated to models and algorithms that build up more complex motion representations, from the prior stage’s processing (Marr and Ullman, 1981; Adelson and Bergen, 1985; Cavanagh and Mather, 1989; Taub et al., 1997; Krekelberg and Albright, 2005; Pack et al., 2006; Tsui and Pack, 2011; Mineault et al., 2012; Krekelberg and van Wezel, 2013; Patterson et al., 2014; for review see Burr and Thompson, 2011). This may be due to the fact that many behavioral and neurophysiological studies of the dorsal stream have used paradigms that are focused on individual motion features instead of object representations. While feature integration and object representations that lead to object based selection are fairly well understood concepts within the context of the ventral pathway, less is known about how and where these processes occur in the dorsal pathway. We will systematically review the studies that do shed light into which stages of the dorsal stream use object representations vs. motion features. Our aims are to provide a framework for object representations within the dorsal stream and propose where the anatomical locations of these representations may be. We find that motion features but not object representations are used up to global motion processing, as is found in area middle temporal (MT). The next stage of processing, area medial superior temporal (MST), relies on intermediate object representations based on smooth pursuit and glass pattern studies. Finally, intermediate object representations can be used by the decision making circuitry further down the dorsal stream (e.g., area lateral intraparietal (LIP)), which results in faster decisions. It should be noted that the review of literature presented here is strictly limited to those processes that are pertinent to the current discussion and thus is not by any means exhaustive.

## Hierarchical visual processing

### Dorsal pathway

**Figure 1:**
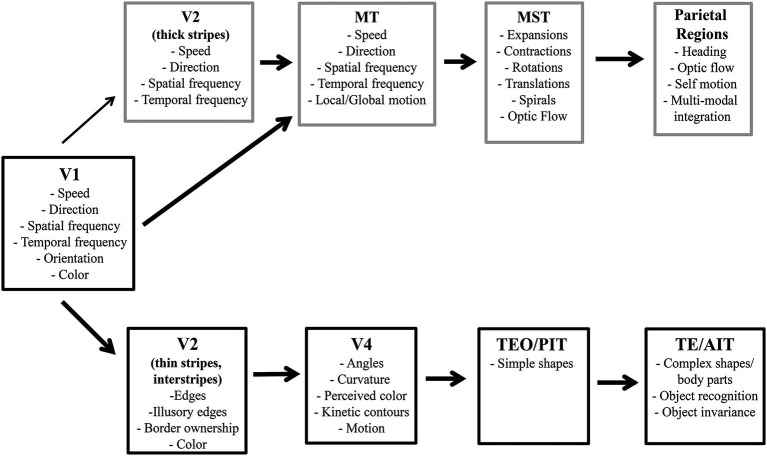
**Hierarchy of visual processing in ventral and dorsal streams**. Gray boxes, from V2 on, depict select features processed at each region along the dorsal pathway. Black boxes, from V2 on, represent features processed along the ventral pathway.

The dorsal visual pathway is specialized for motion processing. Much research has determined the hierarchical nature of motion processing wherein each stage builds upon the previous stage’s output leading to understanding of the algorithms and connectivity to produce models of the different stages of motion processing (Marr and Ullman, 1981; Adelson and Bergen, 1985; Cavanagh and Mather, 1989; Taub et al., 1997; Krekelberg and Albright, 2005; Pack et al., 2006; Tsui and Pack, 2011; Mineault et al., 2012; Krekelberg and van Wezel, 2013; Patterson et al., 2014; for review see Burr and Thompson, 2011). It is important to note that these models focus on the transformation of motion information and not its integration into object representations. Although motion can produce form cues to be used in representing objects in the ventral stream, e.g., structure-from-motion (Johansson, 1973, 1976; Siegel and Andersen, 1988; Bradley et al., 1998; Grunewald et al., 2002; Jordan et al., 2006), object representation in the dorsal stream has not been historically focussed upon. This section briefly reviews the anatomical and functional hierarchy for motion processing (see Figure 1 for an overview).

#### V1

Magnocellular cells in the retina and lateral geniculate nucleus (LGN) provide the input to motion processing in the dorsal pathway. These cells are sensitive to low luminance and also to lower spatial and higher temporal frequencies, but are not sensitive to color. They project to layer 4Cα in the primary visual cortex (V1). In V1complex cells are sensitive to the motion of oriented moving edges, bars or gratings (Hubel and Wiesel, 1968; Hubel et al., 1978; Adelson and Bergen, 1985) and show direction selectivity (Orban et al., 1986; Movshon and Newsome, 1996). Complex cells also show the combined spatiotemporal frequency tuning necessary for early speed selectivity (Orban et al., 1986; Priebe et al., 2006). In addition, it has been shown that V1 cells respond only to the local (or component) motion contained in complex patterns (Movshon and Newsome, 1996).

#### V2

Motion information, from layer 4B in V1, projects to the thick stripes in V2 (Hubel and Livingstone, 1987; Levitt et al., 1994). Although not traditionally thought to play a central role in motion processing, the thick stripes in V2 provide the second largest input to area MT (DeYoe and Van Essen, 1985; Shipp and Zeki, 1985; Born and Bradley, 2005) and it has recently been suggested that directional maps could first emerge in V2 (Lu et al., 2010; however, see Gegenfurtner et al., 1997 for an alternative view).

#### MT

While MT is the next stage of motion processing after V2, it also receives significant input directly from V1 (Felleman and Van Essen, 1991; Born and Bradley, 2005). MT cells are sensitive to many features associated with 2D motion such as direction (Maunsell and Van Essen, 1983; Albright, 1984; Lagae et al., 1993), speed (Maunsell and Van Essen, 1983; Lagae et al., 1993; Perrone and Thiele, 2001; Priebe et al., 2003; Brooks et al., 2011), and spatial frequency (Priebe et al., 2003; Brooks et al., 2011). The increase in receptive field size and the unique characteristics of MT cells allow for the processing of both local (component) and global (pattern/random dot kinetograms) motion (Pack and Born, 2001; gratings: Adelson and Movshon, 1982; Rodman and Albright, 1989; random dot kinetograms (RDKs): Britten et al., 1992; Snowden et al., 1992). This allows MT to both integrate the motion of multiple dots or incongruent motions created by edges within the same object, and also to separate multiple moving objects from each other. It is important to note that neurons in area MT have been shown to not be color selective (Maunsell and Van Essen, 1983; Shipp and Zeki, 1985; Zeki et al., 1991; Dobkins and Albright, 1994; Gegenfurtner et al., 1994).

#### MST

With the local and global 2D motion information from area MT, area MST has been implicated in processing complex, 3D motion and in the start of computations of optic flow and self-motion which are dependent on the analysis of 3D motion. Area MST has been anatomically divided into lateral (MSTl) and dorsal (MSTd) regions, where MSTl is thought to be intricately involved in computing the velocity signals of object trajectories used in the maintenance of pursuit eye movements (Tanaka et al., 1993; Ilg, 2008). In comparison, neurons in MSTd are selective for rotations and expansion/contraction motion (Saito et al., 1986), or their combination, aka spiral motion (Graziano et al., 1994; Mineault et al., 2012). MSTd neurons are also selective for optic flow (Duffy and Wurtz, 1991a,b). In fact MSTd neurons can take optic flow and compute the heading or direction of self-motion (Duffy and Wurtz, 1995; Gu et al., 2006).

#### Beyond MST

After MST, the dorsal pathway continues into the posterior parietal cortex. Motion processing therein involves more complicated optic flow and self-motion patterns, including the motion of objects while the viewer is also moving (Phinney and Siegel, 2000; Raffi and Siegel, 2007; Raffi et al., 2010; Chen et al., 2013; Raffi et al., 2014;). For example, cells in area 7a are tuned to distinguish between *types* of optic flow (Siegel and Read, 1997), and neurons in caudal pole of the superior parietal lobule (Brodmann area 5) (PEc) can combine optic flow information with signals regarding the position of the head and eye (Raffi et al., 2014).

### Ventral pathway

The ventral visual pathway processes form and color information in a hierarchical stream that builds up separately and then integrates into intermediate and full object representations (Marr and Nishihara, 1978; Biederman, 1987; Biederman and Cooper, 1991) ending with object recognition (Cowey and Weiskrantz, 1967; Gross et al., 1971, 1972; Dean, 1976). Thus, hierarchical models of the object representation and recognition focus on feature integration in the ventral stream (SIFT: Lowe, 1987; Neocognitron: Fukushima, 1975; HMAX: Riesenhuber and Poggio, 1999, and others. For review see Poggio and Ullman, 2013). This section briefly reviews the anatomical and functional hierarchy for building up an object in the ventral pathway (see Figure 1 for an overview).

#### V1

Input to V1 in the ventral pathway comes mainly from the parvocellular layers of the LGN with additional magnocellular input (Ferrera et al., 1992, 1994). Parvocellular cells, sensitive to color, high contrasts, and high spatial and low temporal frequencies, project to layer 4Cβ of V1 which is subsequently divided into color blobs and form interblobs. Blobs are color selective but contrast and size invariant (Solomon et al., 2004; Solomon and Lennie, 2005), and untuned for orientation (Livingstone and Hubel, 1987; Ts’o and Gilbert, 1988; Roe and Ts’o, 1999; Landisman and Ts’o, 2002; Shipp and Zeki, 2002). Interblobs are orientation selective for multiple stimulus types, i.e., edges, bars, gratings (Hubel and Wiesel, 1968; Hubel et al., 1978). Both blobs and interblobs process features without regard to objects, although feedback can produce object-based modulation (Roelfsema et al., 1998) and may be involved in representing objects (Fallah and Reynolds, 2001; Roelfsema and Spekreijse, 2001).

#### V2

While color processing (interstripes) changes little from that seen in V1, there is notable progression in form processing (thin stripes). V2 neurons are sensitive to the orientation of edges that are defined either by illusory contours or texture (von der Heydt et al., 1984; Peterhans and von der Heydt, 1989; von der Heydt and Peterhans, 1989). V2 cells also encode border ownership (Zhou et al., 2000) which is the first stage of assigning an oriented edge to an object representation. Thus contour-based object representation starts in V2.

#### V4

Neurons in V4 are tuned for hue that is unaffected by luminance and not limited to a set of colors along the cardinal color axes (red-green, blue-yellow) as seen in V1 (Conway and Livingstone, 2006; Conway et al., 2007). Center-surround interactions produce encoding of perceived color instead of physical color (Schein and Desimone, 1990). Thus, V4 is the first representation of perceived color which is the earliest stage at which color should be incorporated into an ecologically valid object representation.

Form processing in V4 combines multiple, spatially-adjacent, orientation responses seen in V1 and V2 to encode angles and curvatures (Pasupathy and Connor, 1999). These responses advance the nascent object representation from border ownership (Orban, 2008) to responses that are dependent on the placement of the curvature with respect to the center of the shape (Pasupathy and Connor, 2001).

Selection for the orientation of contours created between moving objects (kinetic contours) emerges in V4 (Mysore et al., 2006). Accordingly, a subset of V4 neurons are directionally selective (Ferrera et al., 1992, 1994; Li et al., 2013). Therefore, it should be noted that the intermediate object representations in area V4 can include motion features as well as color and shape.

#### IT cortex

Inferior temporal (IT) cortex has a range of object property complexity starting with simpler features posteriorly (PIT or TEO: Tanaka et al., 1991; Kobatake and Tanaka, 1994) that increase in complexity as processing moves anteriorly (AIT or TE) to represent objects and perform object recognition (Cowey and Weiskrantz, 1967; Gross et al., 1971, 1972; Dean, 1976). This includes complex shapes, combinations of color or texture with shape (Gross et al., 1972; Desimone et al., 1984; Tanaka et al., 1991), and body parts (faces or hands: see Gross, 2008 for a review). In addition, responses in IT cortex are position and size invariant (Sato et al., 1980; Schwartz et al., 1983; Rolls and Baylis, 1986; Ito et al., 1995; Logothetis and Pauls, 1995) and also invariant to changes in luminance, texture, and relative motion (Sáry et al., 1993). Combined, these characteristics make IT ideal for representing objects despite changes in the surrounding environment and retinal image.

## Feature integration in the dorsal stream

Classically, as presented above, it is thought that the ventral pathway is involved in the creation of object representations and categorizations that allow for recognition, object-based selection and decision making processes. Comparatively, the early dorsal stream is most often thought to be specialized for motion processing. Growing evidence suggests however, that processing in the dorsal stream may also allow for object based selection and decision making, which is consistent with later dorsal stream involvement in visumotor guidance, e.g., vision for action (Goodale and Milner, 1992; Goodale, 2008, 2013). In the ventral stream, the object-file theory (Kahneman et al., 1992) has been supported by growing empirical evidence (Mitroff et al., 2005, 2007, 2009; Noles et al., 2005). Object-files collect, store and update information regarding specific objects over time. They are considered to be mid-level representations of objects that do not rely on higher-level object categorizations.

While motion processing studies have focused on individual motion features like direction or speed discriminations of a single moving stimulus, these motion computations could instead be working on intermediate object representations. We hypothesize that later dorsal stream processing occurs on intermediate object representations formed by feature integration instead of on independent motion features. Further we propose that the intermediate object representations also integrate ventral stream information such as color or form. Here we present evidence that support the presence of intermediate (or mid-level) object representations in the dorsal stream, resulting from both ventral and dorsal stream features being integrated into an object-file.

There are multiple ways to investigate the mechanism and timing of feature integration (Cavanagh et al., 1984; Kahneman et al., 1992; Croner and Albright, 1997; Mitroff et al., 2005; Bodelón et al., 2007; Perry and Fallah, 2012 among others). To study feature integration in the dorsal pathway, it is practical to utilize stimuli that activate motion processing regions. Area MT is well known to be involved in direction computations of moving stimuli including the global motion of RDKs (Britten et al., 1992; Snowden et al., 1992). The use of coherently moving, superimposed RDK’s that produce the perception of two superimposed objects moving in different directions controls for spatial location, allowing for investigation of object properties (Valdes-Sosa et al., 1998; Rodríguez et al., 2002; Mitchell et al., 2003; Reynolds et al., 2003; Stoner et al., 2005; Fallah et al., 2007; Wannig et al., 2007). In addition, direction discrimination of two superimposed surfaces becomes more difficult as the presentation time decreases (Valdes-Sosa et al., 1998), suggesting that there is a limitation in speed of processing.

Using two superimposed RDKs does, however, create a perceptual illusion known as direction repulsion. Instead of the directions of the two superimposed surfaces being integrated, the directions are perceived as being repulsed away from the real directions of motion (Marshak and Sekuler, 1979; Mather and Moulden, 1980; Hiris and Blake, 1996; Braddick et al., 2002; Curran and Benton, 2003). This phenomenon can also be observed with superimposed gratings under conditions that produce motion transparency (Kim and Wilson, 1996). Direction repulsion is the result of inhibitory, repulsive interactions (Marshak and Sekuler, 1979; Mather and Moulden, 1980; Wilson and Kim, 1994; Kim and Wilson, 1996; Perry et al., 2014) between the directions of motion at the level of global motion processing in area MT (Wilson and Kim, 1994; Kim and Wilson, 1996; Benton and Curran, 2003). We will present studies on the integration of features into the dorsal stream wherein the direction repulsion paradigm is used to distinguish between perceptual alterations in the magnitude of direction repulsion and processing speeds needed to make the perceptual decisions (Perry and Fallah, 2012; Perry et al., 2014). The results provide insight into where features are integrated and when an intermediate object representation is likely to occur.

### Integration of color

Color is a feature that is processed in the ventral stream through input from parvocellular cells.

Many neuronal studies have found that neurons in the dorsal pathway are not sensitive to color (Maunsell and Van Essen, 1983; Shipp and Zeki, 1985; Zeki et al., 1991; Dobkins and Albright, 1994; Gegenfurtner et al., 1994). In fact, ecologically speaking, color is an irrelevant feature when it comes to processing motion, as in the color of a ball should not matter when attempting to catch it. In spite of this, a number of studies have found that color does in fact alter different aspects of motion processing (Croner and Albright, 1997, 1999; Tchernikov and Fallah, 2010). This would suggest that there is integration of color with motion information in the dorsal stream.

**Figure 2:**
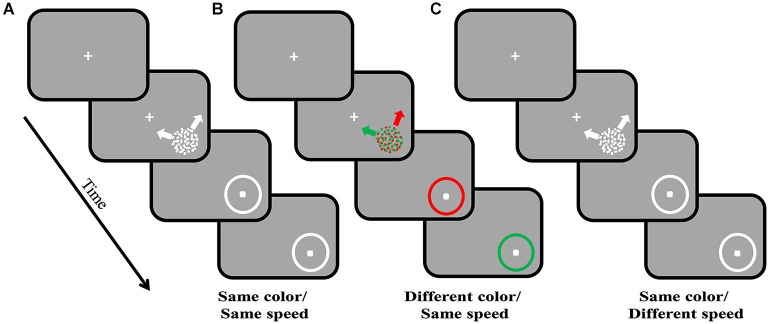
**Direction repulsion staircase paradigm**. Each trial commences with the appearance of a centrally located fixation point. Once fixation is maintained for 200 ms, the visual stimulus, two superimposed, coherently moving in different directions, random dot kinetograms (RDK’s), are presented in the lower right quadrant. In **(A)** the two surfaces are the same color and move at the same speed. In **(B)** the surfaces are the same speed but are different colors, and in **(C)** the surfaces are the same colors but different speeds. The two surfaces are presented for a variable amount of time (staircase procedure). Once they are removed, participants use a mouse to indicate the two directions of motion by clicking on the response circle, once for each direction. In **(B)** participants are required to give the direction for the indicated colored surface in order; the order is randomly assigned between trials. Initially the visual stimulus is presented for 2000 ms, and based on participant’s ability to correctly determine both directions of motion, this time will either increase or decrease in successive blocks of trials. Once participants reach a double reversal of presentation times, the time needed to process both directions of motion can be estimated to within ±50 ms. Direction repulsion is calculated as the difference between the angle created by the two clicks on the response circle and the angle created by the two real directions of motion.

We investigated the effects of color on direction repulsion (Figure 2) to determine whether cross-stream feature integration affects direction discrimination, which would support the use of intermediate object representations in motion processing. Two superimposed, coherently moving RDK’s were presented, initially for 2000 ms. Each surface could move in one of 12 directions relative to either the vertical or horizontal axes, and both directions created angle differences between the two surfaces ranging between 70° and 110°. If participants correctly determined the directions of both surfaces ≥7/8 times, the presentation time decreased, if participants failed to meet this criterion, the time increased. This process continued until participants completed a double reversal. The time needed to process both surfaces correctly (Presentation Time) was estimated to within ±50 ms. Direction repulsion was calculated as being the angle difference between the perceived directions of motion and the actual directions of the surfaces.

If segmenting the two superimposed surfaces by color (Figure 2B) reduced direction repulsion, compared to when both surfaces were the same color (Figure 2A), this would suggest that color information from the ventral stream is integrated into motion processing in the dorsal stream prior to or at the time that global motion processing is computed, e.g., the stage where mutual inhibition gives rise to repulsion.

Previous work found that when segmenting coherently moving dots of one color from distractor dots of a different color in the same RDK, color acts as a filter that allows for improvements in direction discriminations, behaviorally in humans and animals (Croner and Albright, 1997) and in the responses of area MT neurons (Croner and Albright, 1999). In this case, color would be gated earlier (in V2) allowing for the suppression of distractor colored input to MT. This effectively allows MT to process the coherently moving dots as if they were appearing alone and in turn improves direction computation. Thus when the distractor color is known, color filters can suppress input to motion processing, a finding that has been replicated in superimposed surfaces (Wannig et al., 2007). Based on these findings, we hypothesized that integrating the color with the motion of the two superimposed surfaces might also allow for the surfaces to be individually filtered by color and in turn reduce direction repulsion.

**Figure 3:**
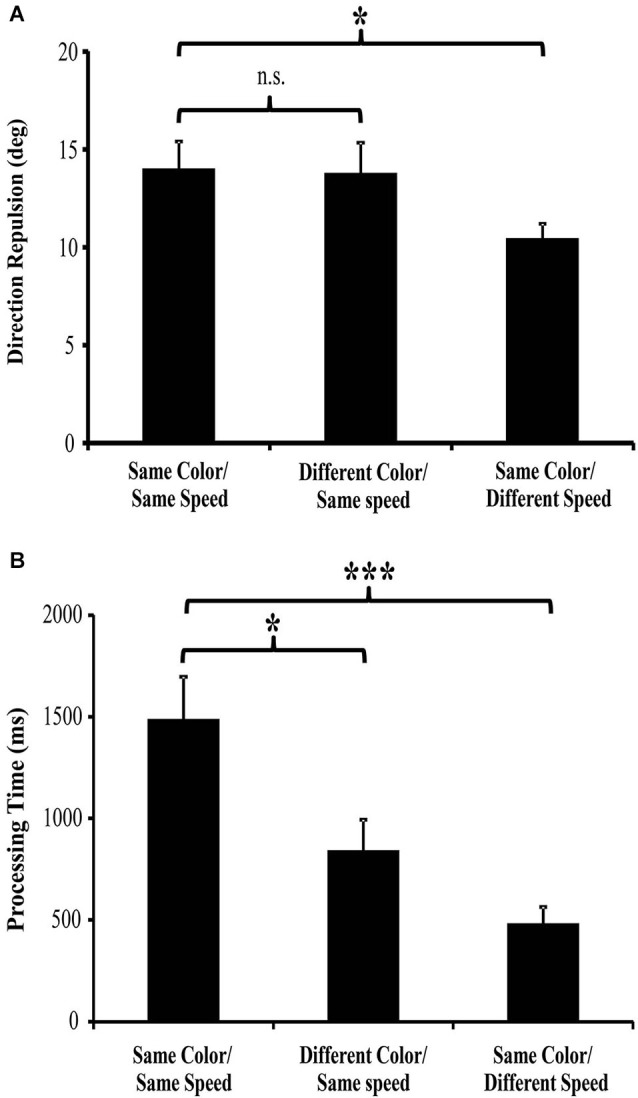
**Direction repulsion and processing time results (from Perry and Fallah, 2012; Perry et al., 2014). (A)** There was no significant modulation of direction repulsion with the addition of color (Different Color/Same Speed: 13.79° ± 1.54 SEM) when compared to the Same color/Same speed (14.02° ± 1.39 SEM) condition. However, direction repulsion in the Same color/Different speed condition (10.47° ± 0.74 SEM) was significantly less than in the Same color/Same speed condition. **(B)** Processing time in both the Different color/Same speed (842 ms ± 209 SEM) and Same color/Different speed (483 ms ± 81 SEM) conditions was significantly less than in the Same color/Same speed (1488 ms ± 208 SEM) condition.

**Figure 4:**
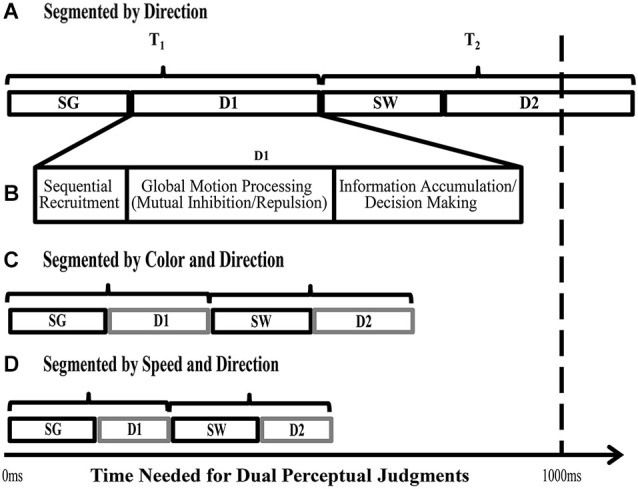
**Stages required for direction judgments of two superimposed objects**. Based on the task described in Figure 3. SG = time needed for *Segmentation* of the two fields of dots into two surfaces, based on different directions of motion, SW = time needed to *Switch* processing from one surface to the other, D1 and D2 = the time needed to process the *Directions* of each superimposed surface (includes sequential recruitment, global motion computation, information accumulation and decision making; shown in detail in **(B)**. **(A)** When the two surfaces differ only in direction, the time needed to complete all the stages involved in the task takes more than 1000 ms on average (Perry and Fallah, 2012). **(B)** Depicts the processes needed to determine the direction of motion of one surface (D1). **(C)** When the surfaces differ in color as well as direction, processing time significantly decreases to less than 1000 ms (Perry and Fallah, 2012). **(D)** When the surfaces differ in speed as well as direction, the time needed to process both directions is reduced further. As the initial segmentation (SG) and attentional switch time (SW) do not appreciably decrease with additional distinguishing features, we propose that the time needed to complete the task decreases as a result of speeded decision making processes (D1 and D2—see text for details) and correspondingly, in **(B)** and **(C)** D1 and D2 are depicted as requiring less time than in **(A)** (adapted from Perry and Fallah, 2012).

Surprisingly, when selecting between multiple moving surfaces that are different colors, direction discrimination is unchanged from that seen when both surfaces are the same color (Figure 3A). Therefore, the global motion processing of a moving RDK is not performed on intermediate object representations, but instead relies on processing the individual motion features. There is however, a large decrease (43% reduction) in the processing time needed to correctly determine both directions of motion. When both surfaces are the same color, processing both directions took almost 1500 ms, but when the surfaces were different colors, processing time was reduced to ~840 ms (Figure 3B; Perry and Fallah, 2012). We have suggested previously (Perry and Fallah, 2012; Perry et al., 2014) that it is most likely processing time is reduced through increasing the speed of the decision making process. Figure 4A depicts the steps necessary to perform the task of judging the directions of two superimposed surfaces, and the time needed for each step (Perry and Fallah, 2012). The superimposed dot fields are first segmented (SG) into two surfaces, and then the direction of one surface is processed (D1). This would include (Figure 4B) sequential recruitment (Nakayama and Silverman, 1984; McKee and Welch, 1985; Mikami et al., 1986), global motion processing, mutual inhibition (repulsion), and information accumulation for decision making (Shadlen and Newsome, 1996; Huk and Shadlen, 2005; Palmer et al., 2005; Zaksas and Pasternak, 2006; Hussar and Pasternak, 2013). Attention is switched (SW) to the second surface, and then the direction of the other surface is processed (D2). When both surfaces are the same color, correctly processing the direction of both surfaces takes more than 1000 ms (Figure 4A), but when the surfaces are segmented by color, the direction of both surfaces is correctly processed in under 1000 ms (Figure 4C; Perry and Fallah, 2012), a ~650 ms decrease in processing time. It could be that the time needed to segment (SG) the two surfaces is reduced when each surface is a different color. However, as segmentation is speeded by not more than 25 ms in texture-defined objects (Caputo and Casco, 1999) this is unlikely the sole mechanism underlying such a large decrease in processing time. Alternatively, switching attention (SW) between the two surfaces may be speeded when each surface is a different color. Switching attention between serially presented objects in the same location (as in attentional blink) requires only a few hundred milliseconds (Raymond et al., 1992)—but can be attenuated by around 100 ms when targets and probes are less similar (Raymond et al., 1995). Again this mechanism is not sufficient by itself to produce the decrease in processing time. Therefore, there must be a reduction in the time needed to process each direction for such a large decrease in processing time to occur.

**Figure 5:**
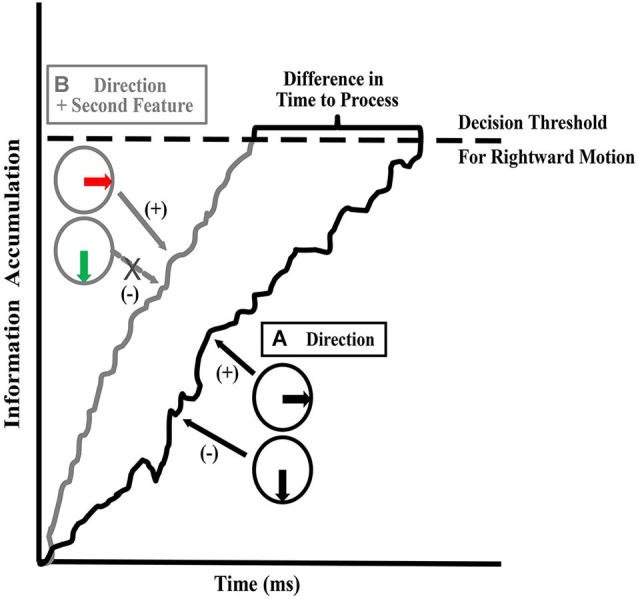
**Information accumulation and decision threshold**. Hypothesized stage at which processing time is reduced. Areas downstream of MT accumulate motion information in order to arrive at a decision. The figure depicts information accumulation for the rightward direction. When accumulating evidence in support of the rightward direction (+), the evidence is reduced by noise created by the presence of the other surface (−). **(A)** When only direction (one feature) differs between the surfaces, interference between the directions of each surface creates a noisy walk: i.e., incongruent input that reduces the accumulated evidence for the rightward direction. This extends the time needed to reach the decision threshold. **(B)** When direction and a second feature such as color or speed differs between the surfaces, the second feature can be used to reduce the interference caused by the other surface (by allowing competitive selection to override the influence of the second surface) in the walk to threshold, thus reducing the time needed to reach a decision threshold.

In order for color to reduce direction processing time (Figure 4C), color input would likely have to affect either the sequential recruitment or decision-making mechanisms including information accumulation (Figure 4B) since it does not affect global motion processing (the mutual inhibition circuit). First, MT needs to associate individual dots across two frames (sequential recruitment: Mikami et al., 1986) and pool that information across enough dots (Britten et al., 1992; Snowden et al., 1992) to determine the global motions of the two surfaces. If color worked on sequential recruitment processes, each dot would only need to be compared to dots of the same color across frames, reducing the possibilities by half, speeding up the process immensely. However, by acting as a color filter on sequential recruitment, this color filtering would also be expected to reduce the direction repulsion illusion as each set of colored dots would be processed individually as described earlier (Croner and Albright, 1997, 1999). Instead, there was no change in direction discrimination when two moving surfaces were superimposed (Perry and Fallah, 2012) which indicates that color could not be used to filter out the second surface and reduce the possibilities during sequential recruitment. Alternatively, the integration of color with motion could affect decision-making. Direction discriminations take the information from motion processing in area MT (Albright, 1984; Mikami et al., 1986; Newsome and Paré, 1988; Salzman et al., 1992), and pass it downstream, to areas like LIP, where it is accumulated and a decision threshold reached (Shadlen and Newsome, 1996; Huk and Shadlen, 2005; Zaksas and Pasternak, 2006; Hussar and Pasternak, 2013). If the two surfaces are identical except for their direction of motion, the direction of each surface interferes with the accumulation of direction information for the other surface (Figure 5A—Palmer et al., 2005). This interference results in a noisy walk to the decision threshold (accumulator model—Palmer et al., 2005). That is, a decision-making neuron accumulating information to make a decision of rightward motion, would treat input from directional cells preferring rightward motion as positive evidence towards reaching threshold, but input from cells preferring downward motion interferes reducing the accumulated evidence. This produces a noisy walk to threshold. More positive evidence would need to be accumulated before threshold is reached, which means more processing time is needed. With a second feature (color) added to each surface, the two sources of input can be distinguished and selected between. This selection can reduce or eliminate the input from the interfering surface, which reduces the noise in the walk towards the decision threshold, increasing the slope and thus reducing processing time (Figure 5B). Therefore, this requires that the accumulation of information for direction discrimination works on intermediate object representations in which color is integrated with motion. This intermediate object representation gives the advantage of allowing for competitive selection of objects (e.g., biased competition: Desimone and Duncan, 1995; Desimone, 1998; Reynolds et al., 2003; Fallah et al., 2007) at later stages of dorsal stream computations such as decision making.

In summary, changes in processing time, due to speeded decision making processes (as proposed above), with no alteration in direction discrimination, suggest that color is integrated into dorsal stream intermediate object representations after global motion processing. This allows for decision-making processes to use those object representations to reach decision thresholds faster.

### Integration of speed

Unlike with color, previous investigations of direction repulsion have shown that when two superimposed surfaces are of different speeds (Marshak and Sekuler, 1979; Curran and Benton, 2003; Perry et al., 2014) or different spatial frequencies (Kim and Wilson, 1996), direction discrimination improves; direction repulsion is attenuated. Given that spatial frequency, speed and direction are all co-processed within MT (Maunsell and Van Essen, 1983; Albright, 1984; Lagae et al., 1993; Perrone and Thiele, 2001), this is perhaps not surprising. Comparison of movement between two frames give us all three of these features. The spatial location of an object from one frame to the next can be used to calculate direction and speed, while spatial frequency can be extracted from the number of times an object appeared over a given distance. So this information comes in together as a single input and does not require integration; it is inherent based on the movement of the stimulus. Consistent with this, neurons in MT are simultaneously selective for multiple motion features, such as speed and direction. Consequently, a neurons response to one feature (direction for example) can be altered by the response of that same neuron to a different motion feature (such as speed), and as a result can be considered to be conjoined, i.e., the processing of one feature affects processing of a different feature (Maunsell and Van Essen, 1983; Albright, 1984; Lagae et al., 1993; Perrone and Thiele, 2001). Based on co-processing, motion processing is reflective then of the presented combination of conjoined features. This occurs without the need for a bound object representation. For example, perception of speed can be distorted under a number of different viewing conditions (Krekelberg et al., 2006a,b). A reduction in contrast reduces perceived speed in slow moving stimuli (Thompson, 1982) and increases perceived speed of fast moving stimuli (Thompson et al., 2006). Perceived speed is also dependent upon spatial frequency (Priebe et al., 2003). And finally the perception of direction is sensitive to motion processing conjunctions: direction discrimination becomes more accurate when superimposed surfaces are different speeds (Marshak and Sekuler, 1979; Curran and Benton, 2003; Perry et al., 2014) or different spatial frequencies (Kim and Wilson, 1996).

These examples suggest that direction computation occurs on conjoined dorsal stream features such as direction and speed or direction and spatial frequency information. Using the same paradigm as described in section Integration of color, but with surfaces that are segmented by differences in speed (Figure 2C), we tested whether speed, while conjoined with direction for discrimination, could also be used as a distinguishing feature in intermediate object representations like color is (Section Integration of color) and similarly speed up decision making circuitry (Perry et al., 2014). As with color (Perry and Fallah, 2012), we found that differences in the speeds of two superimposed surfaces decreased processing time (Figure 3B). In fact, processing time was lower than that seen when the surfaces were segmented by color (Speed-segmented: 483 ms vs. Color-segmented: 841 ms). It could be that velocity, conjoined speed and direction, is the signal that becomes a part of the object representation. If that were the case however, processing time would not be altered as velocity would comprise a single object feature and there would be no other independent feature for use by selection mechanisms to reduce the noise in the walk to threshold (Figure 5) and reach a decision threshold more quickly. Instead these results suggest that speed information is treated as an independent feature in an intermediate object representation that is used by decision making circuitry to speed processing times (Figure 4D; Perry et al., 2014) similar to the effect of color (Perry and Fallah, 2012). Independent in this case simply means that in spite of the fact that speed is co-processed with direction, and their conjunction attenuates direction repulsion during direction computations, speed *alone* can be utilized as a distinguishing feature to select between the object representations when accumulating information for the perceptual decision.

**Figure 6:**
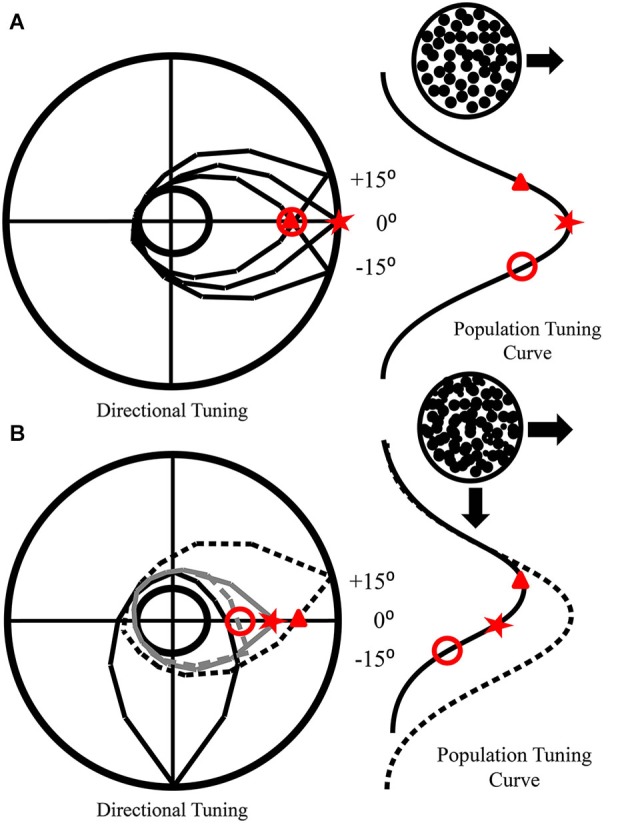
**How mutual inhibition produces direction repulsion. (A)** Individual tuning curves of neurons preferring the direction of motion (0°) and nearby directions (±15°) are presented in the polar plot. The population tuning curve that arises from their responses to rightward motion is also depicted. **(B)** The addition of a second surface moving downwards produces inhibition of other directional neurons. This inhibition drops off as the difference in preferred directions increases. Hence, the −15° neuron is more strongly inhibited (gray dashes) than the 0° neuron (solid gray), while the +15° neuron (black dashes) is not inhibited. This produces a population tuning curve that is shifted away (solid black) from the real direction of motion (dotted line). As the inhibition is mutual, a similar shift would occur for neurons responding to downward motion.

**Figure 7:**
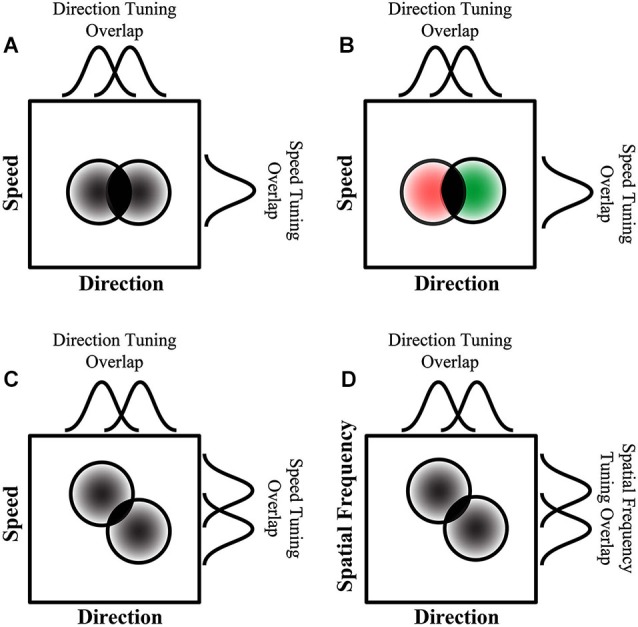
**How additional features affect mutual inhibition and direction repulsion. (A)** Two surfaces that only differ in direction produce direction repulsion whose magnitude is dependent on the area of overlap between their tuning curves (directional tuning curves—top, and two dimensional tuning curves—circles, overlap depicted in solid black). **(B)** When the surfaces are different colors, there is no change in the direction tuning curve overlap which is consistent with color not affecting direction repulsion. However, when a second motion feature that is co-processed with direction, such as speed **(C)** or spatial frequency **(D)**, the population of neurons responding to each direction is segregated based on both features and as a result there is a reduction in the two-dimensional tuning curve overlap (solid black overlap in circles) which results in attenuated direction repulsion (overlaps in **(C)** and **(D)** are smaller than in **(A)** and **(B)**. **(A–D)** The circular plots represent multi-dimensional tuning, while the curves above and to the right of each plot represent the tuning in each dimension respectively (adapted from Perry et al., 2014).

Unlike the effects of color integration, speed differences reduced direction repulsion which further supports that direction discrimination is modulated by other motion features that are conjoined (processed together) in the dorsal pathway. However, ventral stream features, such as color, do not affect motion until after global motion processing occurs. It has been suggested (Marshak and Sekuler, 1979; Mather and Moulden, 1980) that direction repulsion arises due to inhibitory interactions between populations of neurons, a theory recently formalized (Figure 6—adapted from Perry et al., 2014). In mutual inhibition, the responses of neurons to one direction are inhibited by the responses of neurons to a second direction (Figures 6A,B) and the amount of inhibition determines the magnitude of direction repulsion. As the angle between the two directions increases, direction repulsion diminishes (Marshak and Sekuler, 1979; Mather and Moulden, 1980) which suggests that mutual inhibition is dependent upon the overlap in tuning between the neurons responding to the two directions (Figures 6A,B). When the surfaces are identical except for direction (Figure 7A) mutual inhibition and direction repulsion is based solely on the overlap between the tuning curves. Since color is not integrated into motion until after this computation, differences in color do not change the overlap between the two populations and direction repulsion is unaffected (Figure 7B). However, when the surfaces are segmented by dorsal stream features such as speed (Figure 7C) or spatial frequency (Figure 7D) the overlap is reduced due to tuning in multi-dimensional feature space and direction repulsion is decreased. Dorsal stream features are conjoined to produce multi-dimensional tuning and thus do not require integration into an object representation. This is supported by the fact that color, which is part of the object, does not affect this circuitry (Figure 7B). Overall, as direction repulsion is thought to arise from a local circuit in area MT governing global motion processing, the formation of an intermediate object representation that includes speed and color information likely occurs after that stage.

### Integration of form

Artists have long known how to depict motion in still images using features such as speed-lines (the “wake” of a moving object). These non-moving streaks have been shown to affect human perception of motion (Geisler, 1999; Burr and Ross, 2002) by providing a direction input along the orientation of the streak which can either enhance discrimination of a congruently moving stimulus or interfere with incongruent or orthogonal direction discrimination. This motion streak effect is thought to occur as early as V1, supported by computational (Geisler, 1999) and neurophysiological (Geisler et al., 2001) studies. Thus, speed-lines affect the perception of direction by, in effect, producing motion input for use along the dorsal stream. Similarly, glass patterns, paired dots that appear and disappear randomly on a display, give rise to the perception of bistable directions of motion along the contour of the pattern in the absence of underlying motion signals (Glass, 1969; Ross et al., 2000). These spatial patterns produce motion signals that are represented along with magnocellular motion signals in area MT and ST (Krekelberg et al., 2003), and integrate with real motion signals in perceiving direction (Burr and Ross, 2002).

In essence, these form inputs to the dorsal stream provide the equivalent of motion input to mid-level areas in the dorsal stream starting in area MT (Krekelberg et al., 2003). It is likely that the motion produced by these form inputs is then integrated into the object file as motion features (speed, direction) instead of form features. These effects differ from color which is integrated as its own feature into an intermediate object representation later in the dorsal stream hierarchy. That still leaves an open question as to whether other ventral stream features that do not give rise to the perception of motion could also be integrated into dorsal stream object files. Other features could be tested with the same direction repulsion paradigm as described earlier. For example, direction repulsion and processing time could be determined for surfaces distinguished by different contrast levels. As the dorsal stream saturates at much lower contrast than the ventral stream (Heuer and Britten, 2002), if decision-making processing time is affected by contrast differences that are above the saturation point for the dorsal stream, then the dorsal stream object file integrates ventral stream contrast information. Additionally, would a size difference between the dots of the two surfaces result in speeded perceptual decision-making similar to the effects of color? The effects of shape (varying the form of the RDK elements, i.e., dots vs. squares vs. triangles) also needs to be tested.

## Intermediate object representations in the dorsal stream

Thus far, the evidence presented suggests two main concepts. First, global direction computations are based on the co-processing of dorsal stream motion information. Surfaces segmented by speed or spatial frequency (but not color) result in an improvement in direction computations and thus an attenuation of direction repulsion. Secondly, both speed and color are integrated into a dorsal stream intermediate object representation (or object file) which in turn is used by decision making processes to speed processing times. Speed and direction would need to be independent features in a dorsal stream object file, because this allows for awareness of changes in one dimension independent of the other velocity feature. For example, a moving ball provides velocity information (conjoined speed and direction). If it changes speed but continues to move in the same direction, the population of MT cells that would respond to the conjoined speed/direction selectivity changes. Without independence of these motion features in the object representation, switching underlying MT populations would mark a change in all of the conjoined features. Instead, with independence observers are aware of the speed changing while the direction does not. Thus a dorsal stream object file can denote changes in speed or changes in direction independently. We propose that the dorsal stream object file would also include ventral stream information such as color. Decision-making then works on object files instead of direction information alone, and therefore distinguishing features in the object files can be used to selectively focus decision-making on the relevant direction information.

The features that are placed in the object file are dependent upon which features are important to completing the specified task (Harel et al., 2014). Theoretically then, using the direction repulsion paradigm as an example, task relevant would mean that any feature that distinguished the two superimposed surfaces from each other would be a feature added to the object file. This is what occurred with both speed and color, and therefore it would be logical to extrapolate that other task relevant features would also be included in an object file. We have previously suggested (Section Integration of form) how other form features, such as size, shape and contrast, could be tested for integration into a dorsal stream object file.

We propose that global motion processing occurs on conjoined motion features such as speed and direction, whereas the accumulation of perceptual information to reach a decision is performed on intermediate object representations. While these hypotheses are yet to be directly tested at the neurophysiological level (e.g., in animal models), in the next section we propose the likely neural substrates and dorsal stream areas subserving each of these processes, based on known properties of these areas.

### Possible location of object representations in the dorsal stream

**Figure 8:**
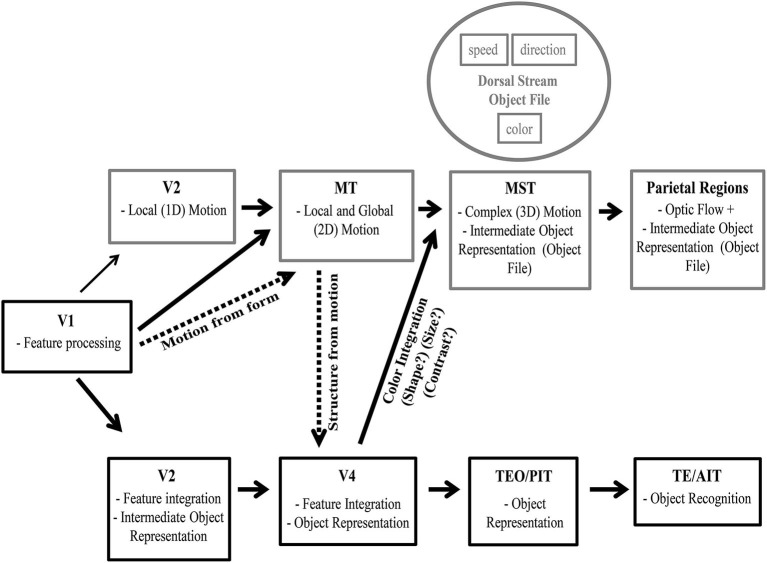
**Intermediate object representation model**. Visual processing along the ventral stream is depicted along with known object representations starting in area V2. We also depict visual processing along the dorsal stream with the hypothetical stages which process dorsal stream object files. As visual processing progresses along the dorsal pathway stimulus parameters are calculated and this information is provided to area MT. In MT, information regarding speed, direction and spatial frequency are co-processed forming multidimensional selectivity. After local and global motion processing circuits in MT, an intermediate object representation is formed that incorporates independent motion features (such as speed and direction) and ventral stream features (such as color, with other features such as shape and size to be determined). This intermediate object representation is in place prior to decision making circuitry that represents motion or guides action.

Figure 8 provides an overview of processing along both the ventral and dorsal pathways with known object representations in the ventral stream and hypothesized object representations in the dorsal stream. Given that object files are considered to be mid-level representations, and are found at intermediate stages of ventral stream processing, they should similarly be found in and around area MT in the dorsal stream. Perceived color is processed in area V4, and thus color processing would need to reach this stage before being incorporated into an object representation in either the ventral or dorsal stream. Color is not integrated with direction prior to direction computation circuits in MT as the addition of color did not reduce direction repulsion. However, color and speed *did* reduce the time needed to fully process both directions of motion. Therefore while global motion direction computations which are computed in area MT are not performed on object files, color and speed are integrated into an object file after direction computation in MT.

Evidence of motion computations relying on object representations comes from smooth pursuit. Color is known to affect smooth pursuit eye movements to moving surfaces (Tchernikov and Fallah, 2010) which are dependent upon the processing of velocity signals for both the surface and the background in area MST (Dürsteler and Wurtz, 1988; Komatsu and Wurtz, 1988, 1989; Thier and Erickson, 1992; Ilg, 2008). Intuitively, eye movements should be color blind. Instead color biases selection of one superimposed surface over the other based on a color hierarchy, and the competition between the two colored surfaces modulates the speed of pursuit (Tchernikov and Fallah, 2010). This suggests that it is not only the reaching and grasping systems later in the dorsal stream that work on object features, but as part of the vision for action pathway, smooth pursuit computations are based on object files. Thus the integration of color into the dorsal stream object file may occur as early as area MST, or at least before the frontal eye fields (FEF) generate the motor plan.

After MST in the dorsal stream, area LIP in the parietal lobe has been shown to be involved in the accumulation of motion information for perceptual decision-making (Shadlen and Newsome, 1996; Huk and Shadlen, 2005; Palmer et al., 2005). This stage of processing works on object files as color and speed differences reduce the time needed to reach the decision threshold. Beyond this stage, a number of areas in the posterior parietal cortex are selective for objects, a function necessary for visuomotor guidance of grasping. Such object selectivity has been found in areas anterior intraparietal (AIP) and 7a (Taira et al., 1990; Murata et al., 2000; Phinney and Siegel, 2000).

This hypothetical framework for object representations in the dorsal stream (Figure 8) can be tested in future neurophysiological studies. Specifically, global motion processing in area MT neurons and the concomitant direction repulsion of the population tuning should not be affected by the addition of color differences. Whereas responses of neurons in area MST that give rise to pursuit motion should be modulated by the color differences in superimposed surfaces (Tchernikov and Fallah, 2010). Finally, decision-making neurons in area LIP should show steeper slopes and reach decision thresholds faster when a second distinguishing feature such as color or speed is present.

### Other evidence for dorsal stream object representations

Other studies have shown selection of objects in the dorsal stream that upon reflection would support intermediate object representations. For example, judging the direction of a brief translation of one of two counter-rotating superimposed surfaces is improved when that surface is selected by color (Valdes-Sosa et al., 2000), an effect the authors attributed to the use of object files by the dorsal stream. The different motions between the two surfaces provides noise in accumulating direction information, but reducing noise through selection of that object file would speed processing such that the decision threshold could be reached during the brief translation period. Similarly, if the object file is selected by a transient motion feature capturing attention, selection of that object file is maintained and again improves the discrimination of a subsequent brief translation (Reynolds et al., 2003) along with modulating the visually evoked N1 component, a marker of selective attention (Pinilla et al., 2001; Khoe et al., 2005). In fact, when one of two superimposed surfaces is selected by a color segmentation cue, the selective advantage for processing brief translations of that surface survives the removal of color differences (Mitchell et al., 2003), once again showing that selection is maintained via an object file. In fact, concurrent judgments of simple form (square or circle) and motion are impaired when made across two superimposed surfaces compared to when they are made for the same surface (Rodríguez et al., 2002). This is similar to Duncan (1984), which showed that attending to an object representation allows judgments of multiple ventral stream form features “for free” but that there was a cost associated with having to make judgments across two superimposed objects. Together, these studies suggest that there are also object representations in the later stages of the dorsal stream. Furthermore, competitive selection processes work not only on objects in the ventral stream (Desimone, 1998; Reynolds et al., 2003; Fallah et al., 2007), but also on objects in the dorsal stream.

### Vision for action

The dorsal stream object representation would not need to progress to the level of object recognition however. As already discussed, the vision for action theory states that the dorsal pathway’s reaching and grasping system uses object features as a means of guiding action in real time. With damage to the ventral stream, patients can still orient their hand and scale their grip according to the orientation and shape of the item to be grasped. This does not require that the object is fully processed through to recognition, just that a list of features associated with a specific object be available for selection (Freiwald, 2007). An object file would provide such a list from which different features could be used to select the correct object among multiple, even superimposed, objects (Valdes-Sosa et al., 1998, 2000; Pinilla et al., 2001; Wannig et al., 2007; Perry and Fallah, 2012; Perry et al., 2014).

### Dorsal to ventral integration

Our proposal is that the dorsal stream integrates features, from both the dorsal and ventral pathways, into an object representation that can be used by decision making circuitry (contained within the dorsal stream) for selection purposes. A similar process occurs in the ventral stream, and it is not only features processed within the ventral stream that are integrated to form object representations used in object recognition and decision making. As early as V4, motion information from the dorsal pathway is used to define stationary edges that occur between moving stimuli (kinetic boundaries—Mysore et al., 2006). However, MT also plays a role in segmentation mechanisms (Born and Bradley, 2005) as a necessary component of surface reconstruction (Andersen and Bradley, 1998). This is what allows MT to separate the motion of multiple moving stimuli from each other (Snowden et al., 1991; Stoner and Albright, 1996), even under conditions of occlusion (Nowlan and Sejnowski, 1995), and to separate moving objects from background (Bradley and Andersen, 1998; Born and Bradley, 2005). Similarly, superimposed dots patterns, moving in opposite directions and moving at variable speeds can be integrated to create a percept of a rotating cylinder. This indicates that processing along the dorsal pathway also allows for perception of 3D structures (Bradley et al., 1998; Dodd et al., 2001). Moving dots are also known to give rise to human shape percepts. Moreover, this perception of biological motion goes beyond shape and form processing. Higher order features, such as gender, are also derived from biological motion (Barclay et al., 1978; Mather and Murdoch, 1994; Jordan et al., 2006). As gender is derived from the global, not local motion, and gender adapts with prolonged exposure to biological motion (Jordan et al., 2006), this occurs at a stage beyond area MT. Biological motion is represented in the superior temporal polysensory area (STP: Perrett et al., 1989) and as such is an object representation later along the dorsal stream, which gives rise to gender representation.

### Alternative location for the object representation

While evidence supports the dorsal stream decision-making processes working on object representations, the site for these representations are unknown. We have suggested that intermediate object representations are built up at later stages in the dorsal stream (Figure 8). However, these decision making circuits in the dorsal stream could instead be modulated by object representations contained in the ventral pathway.

For this to occur, motion information would have to be a tag (e.g., Finger of INSTantiation (FINST): Pylyshyn, 1989, 1994) associated with object processing in the ventral stream, which would then have to be passed back to the dorsal stream in time for direction decisions to be made. While this is possible, Occam’s razor suggests the more parsimonious explanation of dorsal stream object files is likely the correct one. There is a means of testing whether intermediate object representations occur in the dorsal stream. As visual agnosiacs have damage to the ventral stream but retain certain form information used to guide grasps, they could be tested to see whether motion decision-making could be sped up without ventral stream object representations. If so, then there must be dorsal stream intermediate object representations separate from those in the ventral stream. Such intermediate object representations would not give rise to recognition but would incorporate the form features maintained in the dorsal stream to provide real-time visual guidance for actions such as hand orientation, grip scaling, and pincer grip locations (Goodale et al., 1991, 1994; Milner et al., 2012). Note that even if the intermediate object representation was to be created in the ventral stream, it would still be used by decision-making areas in the dorsal stream. The areas that give rise to the object representation would change, but the later stages of dorsal stream processing would still be dependent on object representations, not just motion information.

## Conclusions

We have provided a framework for not only how the dorsal stream extracts motion information but also builds up an object representation that is used in decision making processes. The hierarchical nature of visual processing, in both the ventral and dorsal pathways, provides the basis for where an object representation in the dorsal pathway would exist. Both color and speed information, as independent object features, are integrated into motion processing circuits beyond direction computations (such as in area MT) and prior to decision making and attentional selection (such as in area LIP). In fact, color-dependent smooth pursuit may indicate an intermediate object representation occurs as early as area MST. It is also likely that later parietal areas that guide grasping, such as AIP, may also contain the requisite circuitry for intermediate object representations in the dorsal stream. We have suggested that this object representation would not give rise to object recognition as in the ventral stream but instead would contain a list of object features upon which decisions could be made and actions performed. Object files are a possible mechanism through which information necessary for dorsal stream decision making and selection could be collected and updated as needed. The use of dorsal stream information for the creation of objects in the ventral pathway supports our proposal of parallel mechanisms existing in the dorsal stream. Testing visual agnosiacs on dorsal stream decision making, requiring the use of object representations, would be a way to determine if the dorsal pathway alone can support these intermediate object representations.

## Conflict of interest statement

The authors declare that the research was conducted in the absence of any commercial or financial relationships that could be construed as a potential conflict of interest.
